# Brief periods of transcutaneous auricular vagus nerve stimulation improve autonomic balance and alter circulating monocytes and endothelial cells in patients with metabolic syndrome: a pilot study

**DOI:** 10.1186/s42234-023-00109-2

**Published:** 2023-03-31

**Authors:** Tercio Lemos de Moraes, Fernando Oliveira Costa, Danielly Gomes Cabral, Daniella Marques Fernandes, Carine Teles Sangaleti, Maria Aparecida Dalboni, Josiane Motta e Motta, Liliane Appratto de Souza, Nicola Montano, Maria Claudia Irigoyen, Michael Brines, Kevin J. Tracey, Valentin A. Pavlov, Fernanda M. Consolim Colombo

**Affiliations:** 1 Nove de Julho University – UNINOVE, São Paulo, Brazil; 2 Midwestern State University (UNICENTRO), Paraná, Brazil; 3 Institute of Cardiology of Rio Grande Do Sul, Porto Alegre, Brazil; 4 Department of Clinical Sciences and Community Health, University of Milan, Milan, Italy; 5 University of São Paulo, Hypertension Unit, São Paulo, Brazil; 6 The Feinstein Institutes for Medical Research, Northwell Health, Manhasset, NY USA

**Keywords:** Transcutaneous auricular vagus nerve stimulation, Metabolic syndrome, Cardiovascular autonomic control, Monocyte, Endothelial dysfunction, Inflammation

## Abstract

**Background:**

There is emerging evidence that the nervous system regulates immune and metabolic alterations mediating Metabolic syndrome (MetS) pathogenesis via the vagus nerve. This study evaluated the effects of transcutaneous auricular vagus nerve stimulation (TAVNS) on key cardiovascular and inflammatory components of MetS.

**Methods:**

We conducted an open label, randomized (2:1), two-arm, parallel-group controlled trial in MetS patients. Subjects in the treatment group (*n* = 20) received 30 min of TAVNS with a NEMOS® device placed on the *cymba conchae* of the left ear, once weekly. Patients in the control group (*n* = 10) received no stimulation. Hemodynamic, heart rate variability (HRV), biochemical parameters, and monocytes, progenitor endothelial cells, circulating endothelial cells, and endothelial micro particles were evaluated at randomization, after the first TAVNS treatment, and again after 8 weeks of follow-up.

**Results:**

An improvement in sympathovagal balance (HRV analysis) was observed after the first TAVNS session. Only patients treated with TAVNS for 8 weeks had a significant decrease in office BP and HR, a further improvement in sympathovagal balance, with a shift of circulating monocytes towards an anti-inflammatory phenotype and endothelial cells to a reparative vascular profile.

**Conclusion:**

These results are of interest for further study of TAVNS as treatment of MetS.

**Supplementary Information:**

The online version contains supplementary material available at 10.1186/s42234-023-00109-2.

## Introduction

A cluster of cardiometabolic derangements, including abdominal obesity, hyperglycemia, high blood pressure, and dyslipidemia (high triglycerides, low high-density lipoproteins, HDL) define the metabolic syndrome (MetS), a disorder of pandemic proportions (Grundy et al. 2005; Afshin et al. 2017). MetS is associated with a substantially increased risk for the development of type 2 diabetes, cardiovascular diseases (CVD) and other debilitating disorders (McNeill et al. 2005; Lopes et al. 2016). Although the pathogenesis of the MetS, also referred as Visceral Adiposity Syndrome (Grundy et al. 2005; Afshin et al. 2017), remains incompletely understood, an important underlying event is the infiltration of the expanded visceral abdominal tissue with macrophages and other immune cells (Eckel et al. 2005). The crosstalk between these immune cells, which are of pro-inflammatory phenotype, and enlarged adipocytes with altered metabolic profile results in an increased production of cytokines and a chronic inflammatory state that directly interferes with insulin signaling, resulting in insulin resistance (Lopes et al. 2016; Pavlov 2021). It is also well established that endothelial dysfunction is among the early manifestations of vascular injury in MetS (Deedwania 2003; Kajikawa and Higashi 2022), which amplifies systemic inflammatory responses, creating a vicious cycle that worsens vascular damage (Libby and Hansson 2019). Moreover, patients with MetS with a reduced HDL-cholesterol level present with a significant increase of non-classical monocytes (CD14^+^CD16^+^) and a decrease in classical monocytes (CD14^+^CD16^−^). This change in the subpopulation of monocytes subtypes favors a pro-inflammatory profile (Grün et al. 2018). Hence, the quantification of both circulating monocytes having different inflammatory profiles and endothelial cell lineage, especially endothelial progenitors, could indicate both immune status and early vascular damage in patients with MetS (Deedwania 2003; Kajikawa and Higashi 2022).

Although there is critical need for the treatment of MetS, there are currently no specific therapeutic approaches, which target all components of MetS. Implementation of lifestyle changes, such as exercise and diet, can potentially alleviate MetS constituents, but there are numerous obstacles in adherence to these treatments (Lopes et al. 2016; Eckel et al. 2005). Pharmacological options are mainly directed towards the treatment of individual components grouped in MetS (McNeill et al. 2005; Eckel et al. 2005).

In addition to the chronic low-grade inflammatory state in MetS, preclinical and clinical studies have shown alterations in the autonomic nervous system regulation of visceral adipose tissue metabolism (Pavlov 2021; Chang et al. 2019). Sympathetic overdrive and decreased parasympathetic (vagus nerve) activity are attributed to the metabolic and immune dysregulation in MetS and correlate positively with the development of type 2 diabetes and CVD (Lambert et al. 2010; Schlaich et al. 2015). Research over the last 20 years has revealed the role of the vagus nerve and cholinergic signaling in the regulation of inflammation through a physiological mechanism termed the *inflammatory reflex* (McNeill et al. 2005; Lopes et al. 2016; Eckel et al. 2005). Electrical vagus nerve stimulation (VNS) has an anti-inflammatory and disease-alleviating efficacy in preclinical and clinical settings of numerous inflammatory conditions (Falvey et al. 2022; Clancy et al. 2014; Eberhardson et al. 2020). There is also abundant experimental evidence that administration of drugs that increase cholinergic signaling also suppresses adverse inflammation and metabolic derangements in preclinical and clinical scenarios (Pavlov and Tracey 2017; Pavlov and Tracey 2022; Consolim-Colombo et al. 2017). We have previously demonstrated that galantamine, a centrally acting acetylcholinesterase inhibitor, improves autonomic function, decreases pro-inflammatory cytokines, and alleviates oxidative stress, associated with a reduction in insulin resistance in patients with MetS (Consolim-Colombo et al. 2017; Sangaleti et al. 2021).

In parallel with cytokine production, immune cell migration is modulated by the autonomic nervous system (Pavlov 2021; Deedwania 2003). Previous studies demonstrated that VNS downregulates neutrophil migration and reduces the proportion of circulating pro-inflammatory monocytes in animal models of inflammation (Saeed et al. 2005; Boland et al. 2011). However, the effects of VNS on endothelial and PBMC phenotypes in MetS patients have not been previously characterized. Bioelectronic devices which deliver electrical impulses to activate vagal circuits are currently being utilized in experimental and clinical settings to treat chronic inflammatory conditions and cardiovascular diseases (Kajikawa and Higashi 2022). In addition to the use of VNS through surgically implanted devices, there is a growing interest in using non-invasive approaches such as transcutaneous auricular vagus nerve stimulation (TAVNS) (Kajikawa and Higashi 2022) which activates afferent vagus nerve fibers.

The objectives of the present study were to: 1) investigate the effect of a brief period of TAVNS (1 session of 30 min) on hemodynamic and autonomic parameters in patients with MetS; and 2) examine whether chronic TAVNS (weekly 30 min-session, for 8 weeks) alters circulating monocytes and endothelial cell lineage in patients with MetS.

## Methods

### Study design and participants

This is a randomized, open-label, prospective, interventional, parallel, treatment-controlled study. The researchers who analyzed the data were blinded to the patient’s group allocation. The study is registered at http://www.ensaiosclinicos.gov.br/rg/RBR-7thswq/, with the identifier REQ-7thswq. All patients included in the study provided written informed consent. Patients older than 18 years, both genders, with central obesity, that attended at the UNINOVE MetS Research Outpatient Clinic were invited to participate in the study. Subjects that accepted were initially screened for the inclusion and exclusion criteria.

#### Inclusion criteria

Diagnosis of MetS according to the revised ATP III criteria (Grundy et al. 2005), including three or more of the following five criteria: waist circumference (≥ 102 cm in men, ≥ 88 cm in women),triglycerides (≥ 150 mg / dL); HDL-cholesterol (≤ 40 mg / dL in men, ≤ 50 mg / dL in women); BP (systolic ≥ 130 and / or diastolic ≥ 85 mm Hg); fasting blood glucose (≥ 100 mg / dL); to have normal renal and thyroid function.

#### Exclusion criteria

Patients using insulin and beta-blockers; patients with significant metabolic alteration, such as: HbA1c > 8%; triglycerides (≥ 400 mg / dL); serum levels of alanine transaminase (AST) and / or aspartate transaminase (ALT) ≥ 200U / L; resting systolic blood pressure ≥ 160 mmHg or diastolic blood pressure ≥ 100 mmHg; pregnancy, active neoplasm, skin lesions and / or lack of tactile sensitivity in the left ear.

**Fig. 1:**
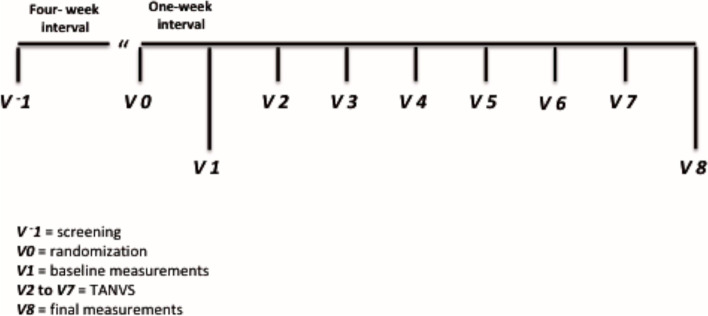
Sequence of the protocol

For the sample calculation we considered a difference of 20% on the LF / HF ratio of the heart rate variability between the groups. We expected a 5% error and 95% confidence interval. Moreover, we aimed to include more patients in the TAVNS arm to evaluate tolerability and adverse events related to the intervention. Therefore, we randomized MetS patients in a 2:1 fashion, *i.e*., we included a total of 30 subjects, 20 subjects were allocated at the treatment group (TG) and 10 at control group (CG). An independent investigator performed random allocation by raffling previously identified cards (10 GC cards and 20 GT cards). The sequence of the protocol is presented in Fig. 1.

At *visit -1*, denominated screening visit, patients were clinically scrutinized for possible inclusion criteria, including a detailed physical examination of the left ear to rule out any outbreak of infection or cutaneous lesion and / or loss of skin sensitivity, the presence of which excluded participation. The anthropometric evaluation included waist circumference, weight, height and BMI calculation. Office blood pressure measurements were performed in a sitting position according to the current guidelines (Flack and Adekola 2020). New fasting blood tests were requested for selected patients with potential to be included in the protocol, to validate inclusion and exclusion criteria, and a return was scheduled in four weeks. At *visit 0* the laboratory results were analyzed. If the patient was eligible for the protocol the randomization was performed, and a return was scheduled in one week to start the protocol (*visit 1*) to perform the baseline measurements. All patients were asked to abstain from exercise for 24 h prior to *visit 1* and from drinking caffeinated products on the morning of this evaluation. All patients were oriented to maintain their regular activity and use of medication during the entire 8-week period. At *visit 1* baseline measurements were performed: a blood sample was withdrawn to quantify peripheral blood cells, hemodynamic and autonomic measurements were collected for all patients, and the first TAVNS was applied in patients allocated at the treatment group. These procedures are described below. At *visits 2*,* 3*, *4*, *5*, *6* and *7* only patients allocated at the treatment group received TAVNS. At *visit 8* other measurements were performed: a blood sample was withdrawn to quantify peripheral blood cells and hemodynamic and autonomic measurements were collected for all patients; the last TAVNS was applied in patients allocated to the treatment group.

### Determination of peripheral monocytes and progenitor endothelial cells

Venous blood samples were withdrawn twice, at baseline (*visit 1*) and the end of the protocol (*visit 8*) into an ethylenediaminetetraacetic acid (EDTA) tube. Considering the group that received TANVS, on visit 1 the blood was withdrawn before the stimulation, and on visit 8, after this procedure. For characterization of monocytes, one hundred (100) μL of EDTA whole blood was incubated with 2 μL of FITC-labeled anti-CD14 monoclonal antibody (classical monocytes) (BD Biosciences, San Diego, CA) and 2 µL of APC Cy7 labeled anti-CD16 monoclonal antibodies (non-classical monocytes) (BD Biosciences, San Diego, CA) for 15 min incubation in the dark at room temperature. After this incubation, the red blood cells were lysed with FACS lysing solution (BD Biosciences) for 10 min and centrifuged at 2000 rpm for 10 min. The supernatant was discarded, and the cells were washed twice with PBS and centrifuged at 2000 rpm for 10 min. The final pellet was resuspended in 300 μL of PBS with 1% sodium azide. The analysis was performed immediately with a flow cytometer BD Accuri (BD Biosciences, San Diego, CA, USA). Forward (FSC) and side scatter (SSC) were used to gate for monocytes to exclude cellular debris (Supplementary Figure 1). For each sample, 30,000 cells were acquired and analyzed. The quantity of CD14 and CD16 expression were presented respectively as percentage from the monocytes gate. Endothelial cells (ECs) were obtained from 5 mL EDTA whole blood from each subject and centrifuged for 5 min at 14,000 rpm to pellet the endothelial cells. The supernatant was discarded, and the ECs were washed twice with PBS and centrifuged at 2000 rpm for 10 min. Then 50 μL from ECs were incubated with 2 μL of monoclonal antibodies against PE-labeled anti- CD31 plus APC-labeled anti- CD144 monoclonal antibody (endothelial microparticles cells/EMCs)) (BD Biosciences, San Diego, CA), according to the manufacturer’s instructions, for 30 min in the dark at room temperature. After incubation, 450 μL of buffered saline solution (HBS; 20 mM HEPES, 150 mM NaCl, 2.5 mM calcium) were added into the tube (ready to analyze). To endothelial progenitor cells, 50 μL from ECs were incubated with 2 μL of monoclonal antibodies against PE-labeled anti-CD309, according to the manufacturer’s instructions, for 30 min in the dark at room temperature. After incubation, 450 μL of buffered saline solution (HBS; 20 mM HEPES, 150 mM NaCl, 2.5 mM calcium) were added into the tube (ready to analyze). For the analyses, we constructed the gate with the FSC and SSC through beads between 100–1000 nm in diameter that is the around the same size of these cells (Supplementary Figure 1). Besides, CD31^+^CD144^+^ expression have been described to enable the characterization of endothelial microparticles by flow cytometry. The analysis was performed immediately with a flow cytometer BD Accuri (BD Biosciences, San Diego, CA, USA). For each sample, 30,000 events were acquired and analyzed. The quantity of endothelial cells (CD31, CD144, CD309) expression were presented as percentage from the endothelial cells gate (Supplementary Figure 1).

### Hemodynamic parameters and heart rate variability (HRV) analyzes

These parameters were recorded at baseline (*visit 1*) and at the end of the protocol (*visit 8*). During the study period, subjects were supine and awake in a quiet room while blood pressure waveforms (BP) were captured and stored using a digital photoplethysmograph device (Finometer, Finapres Medical System BV, Holland) as previously described (Consolim-Colombo et al. 2017). We recorded beat-to-beat blood pressure curves 30 min before and 30 min after the end of stimulation in the treated group. In the control group, a similar recording period was used. Recordings were visually inspected to remove non-stationary data. Sequential pulse intervals (PI) were recorded and then used to compute HRV in both frequency and time domains. All analyses adhered to standards developed by the Task Force of the European Society of Cardiology and the North American Society of Pacing and Electrophysiology Heart rate variability: standards of measurement, physiological interpretation and clinical use. Task Force of the European Society of Cardiology and the North American Society of Pacing and Electrophysiology. (Heart rate variability: standards of measurement, physiological interpretation and clinical use 1996).

### Transcutaneous Auricular Vagus Nerve Stimulation (TAVNS) Protocol

TAVNS was performed using NEMOS device ® (Cerbomed, Erlangen, Germany), a system approved for treatment of epilepsy and depression. This device stimulates the *cymba conchae* of the left outer ear, a region innervated by the auricular branch of the vagus nerve that provides an opportunity to stimulate the vagal afferent pathway to the brainstem (Carnevale et al. 2020; Qureshi et al. 2020). The electrical stimulus used had a pulse width of 500 ms, a pacing frequency of 25 Hz and amplitude in milliampere (mA) ranging from 0.1 to 5.0 mA. Stimulation was performed only in the treatment group during a 30 min period, once a week for the following seven weeks visits 1, 2, 3, 4, 5, 6, 7 and 8.

### Statistical analysis

To establish baseline comparability of the two randomized treatment arms, the chi-square test was used to compare categorical variables (*i.e*., gender) and the Mann–Whitney test, was used to compare continuous measures. Repeated measures analysis of variance (RMANOVA) was used to determine if the two groups behave differently over time (*i.e*., the group x time interaction) for each of the following measures: systolic and diastolic blood pressure, heart rate, low frequency (LF) of HRV, high frequency (HF) of HRV (absolute and normalized values), LF/HF ratio, metabolic parameters, peripheral mononuclear and endothelial cells. For all analyses, the standard assumptions of Gaussian residuals and equality of variance were tested. The repeated within-subjects factor was time (pre- and post), and the within subjects’ factor was treatment group (TAVNS and control). Data analyzed on the raw scale are reported as the arithmetic difference, calculated as post- minus pre-, and standard deviation (SD) for each group. *P* value < 0.05 was considered significant.

## Results and Discussion

**Fig. 2:**
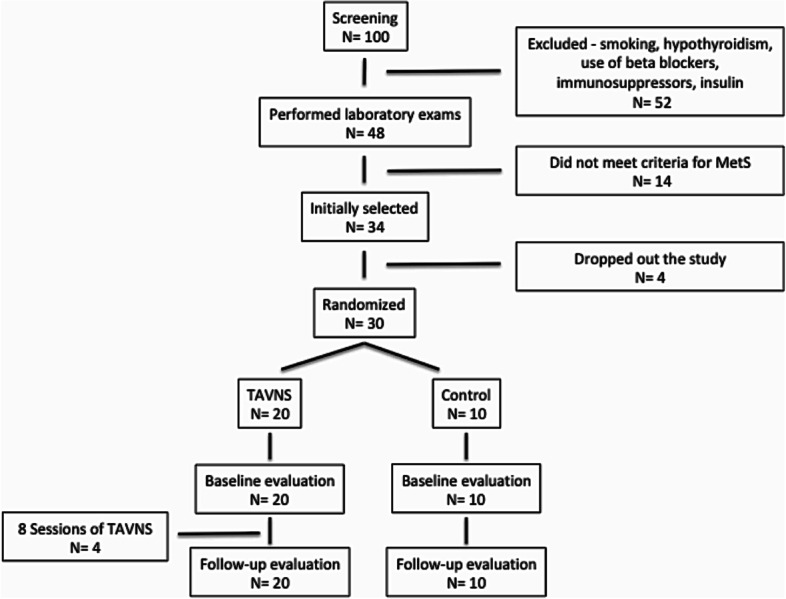
Flow chart of the protocol

Initially one hundred subjects were screened and 52 were excluded considering different causes (smoking, hypothyroidism, use of drugs such as beta-blockers, immunosuppressants, or insulin). A total of 48 subjects underwent new laboratory evaluation, and after the results, 14 did not meet the criteria for diagnosis of MetS. Four patients decided to drop out of the study before randomization. Therefore, 30 patients were included, and all then finished the protocol (Fig. 2).

At the beginning of the protocol, clinical, anthropometric, and laboratory data, did not differ between the two groups (*p* > 0.05) (Table 1).

**Table 1:** Baseline clinical and fasting laboratory profile of patients in control and the TAVNS groups at baseline

	**Control** **(*n* = 10)**	**TAVNS** **(*n* = 20)**	***p***
**Gender (M/F)**	3/7	7/13	
**Age**	46 ± 10	43 ± 9	0.473
**Weight (Kg)**	104 ± 22	105 ± 18	0.960
**AC (cm)**	122 ± 13	119 ± 12	0.544
**BMI (Kg/m2)**	37 ± 6	37 ± 5	0.854
**SBP (mmHg)**	135 ± 21	137 ± 21	0.844
**DBP (mmHg)**	80 ± 10	81 ± 10	0.719
**HR (bpm)**	74 ± 10	72 ± 7	0.659
**Glucose (mg/dL)**	105 ± 17	100 ± 14	0.875
**Total cholesterol (mg/dL)**	201 ± 49	197 ± 42	0.832
**HDL-c (mg/dL)**	48 ± 12	46 ± 10	0.611
**LDL-c (mg/dL)**	125 ± 43	123 ± 39	0.946
**Triglycerides (mg/dL)**	130 ± 42	141 ± 38	0.936
**AST/GOT (U/L)**	24 ± 8	23 ± 9	0.791
**ALT/GPT (U/L)**	28 ± 14	25 ± 7	0.520
**TSH (uIU/mL)**	2.2 ± 1.0	2.3 ± 1.6	0.822
**T4 free (uIU/mL**	1.1 ± 0.2	1.1 ± 0.2	0.697
**Insulin (mUI/mL)**	15 ± 7	17 ± 7	0.438
**CRP (mg/dL)**	0.6 ± 0.4	0.6 ± 0.3	0.916
**HOMA-IR**	3.7 ± 2,0	4.2 ± 1.7	0.754

Values = mean ± SD

*AC* Abdominal circumference, *BMI* Body mass index, *SBP* Systolic blood pressure, *DBP* Diastolic blood pressure, *HR* Heart rate, *HDL* High density lipoproteins, *LDL* Low density lipoproteins, *AST/GOT* Aspartate aminotransferase, *ALT/GPT* Alanine aminotransferase, *TSH* Thyroid-stimulating hormone, *T4* Thyroxine, *CRP* High sensitivity C reactive protein, *HOMA-IR* Homeostatic Model Assessment of Insulin Resistance

### A single session of TAVNS reduces HR and improves sympathovagal balance

One of the objectives of this study was to evaluate the effects of a single TAVNS in patients with MetS, considered an acute effect. Thus, office SBP, DBP and HR measurements and beat-to beat blood pressure curves were registered at baseline and after a TAVNS 30-min session (Table 2). No significant differences in both office and beat-to-beat recording were detected in SBP and DBP measures. However, the office and beat-to-beat analyses of HR showed a small but significant decrease after TAVNS (*p* = 0.022 and* p* = 0.011, respectively).

**Table 2:** Effect of a single TAVNS on hemodynamic parameters and heart rate variability components in MetS patients

	**TAVNS (*n* = 20)**		***p***
	**Baseline**	**After 30 min of TAVNS**	
**Office parameters**			
SBP (mmHg)	137 ± 21	136 ± 22	0.859
DBP (mmHg)	81 ± 10	80 ± 10	0.825
HR (bpm)	72 ± 7	69 ± 8	0.022*
**Beat-to-beat registry**			
SBP (mmHg)	138 ± 20	135 ± 23	0.267
DBP (mmHg)	78 ± 8	78 ± 9	0.755
HR (bpm)	70 ± 8	67 ± 8	0.011*
**HRV**			
VARR	2749 ± 2793	3643 ± 4791	0.192
RMSSD (ms)	41 ± 28	48 ± 42	0.196
LF abs (ms^2^)	772 ± 839	1015 ± 1756	0.347
HF abs (ms^2^)	896 ± 1362	1372 ± 2228	0.149
LF/HF ratio	1.4 ± 0.9	1.1 ± 0.7	0.010*

*SBP* Systolic blood pressure, *DBP* Diastolic blood pressure, *HR* Heart rate, *VARR* Total variance of RR interval, *RMSSD* Square root of the mean of the square of successive differences between adjacent RR intervals, *LF abs* Low frequency spectral power, *HF abs* High frequency spectral power

^*^p < 0.05

TAVNS also caused a significant increase in HF (nu) component suggesting an increase in vagus nerve activity (vagal modulation of the heart), and a decrease in the LF (nu) component suggesting a lower sympathetic activity (data not shown). Consequently, the ratio LF/HF decreased pointing to an acute alteration (improvement) in the autonomic balance (Table 2).

Alterations in autonomic neural regulations, such as increased sympathetic activity and/or reduced parasympathetic (vagus) nerve activity as indicated by HRV are not only a powerful and independent predictor of poor prognosis in patients with cardiovascular disease, but also a risk factor for mortality in healthy populations (Carandina et al. 2021) and MetS patients (Lopes et al. 2016; Eckel et al. 2005; Lambert et al. 2010; Schlaich et al. 2015). Accordingly, using TAVNS to shift HRV towards increased parasympathetic/vagal activity may be beneficial in these disorders. The electrode was placed at the cymba conchae, an area of the outer ear with highest density afferent fibers within the auricular branch of the afferent vagus nerve (Peuker and Filler 2002) which project to the brainstem nucleus tractus solitarius (NTS), thereby providing a physiological substrate for TAVNS efficacy (Yakunina et al. 2017). The decrease in HR observed following TAVNS in the present study might be related to an increase in NTS neuronal activity, which subsequently activates signaling of efferent vagus neurons innervating the heart. In the frequency domain analysis of HRV, involving HF, LF and LF/HR ratio, significant differences were observed in the treatment group, which together confirm that a single session of TAVNS affected cardiac activity through changes in both sympathetic and parasympathetic components.

Robust pathophysiological evidence demonstrates that patients with MetS have an increase in cardiac sympathetic modulation with a greater sympathetic vasomotor drive (Lopes et al. 2016; Eckel et al. 2005; Lambert et al. 2010; Schlaich et al. 2015). Prior studies, which have investigated effects of vagus nerve stimulation on HRV, have generated various results depending on the study design, such as included population, the region of stimulation and its duration, and methods of HRV analysis. Some studies using TAVNS for shorter periods (5 or 10 min) in healthy subjects have also showed significant improvement in HRV parameters of parasympathetic activity (Forte et al. 2022; Geng et al. 2022). However, regarding the LH component, it is suggested that prolonged TAVNS stimulation (30 – 60 min) is required to decrease its power (Tran et al. 2019). Moreover, other studies using a similar protocol in young healthy men showed an increase in LF/HF ratio spontaneous baroreflex sensitivity (Antonino et al. 2017; Clancy et al. 2014). In the present study, we demonstrated an improvement in several autonomic parameters with acute TAVNS in middle-aged MetS patients. Therefore, TAVNS may be an important intervention to rebalance of the autonomic modulation in MetS patients.

### Repeated TANVS decreases blood pressure and heart rate and accentuates changes in HR and HRV without changing metabolic parameters

Comparing clinical data of both groups obtained at baseline (V1) and at 8 weeks of follow-up (V8) revealed a significant decrease in systolic and diastolic blood pressures and heart rate in patients that received TAVNS. No changes were detected in the control group (Table 3). Regarding laboratory evaluation, there were no differences detected comparing baseline and follow-up evaluation in both groups (data not shown).

**Table 3:** Comparison of clinical parameters of the control and TAVNS groups at baseline and after 8-weeks of follow-up

	**Control** **(*n* = 10)**		**TAVNS** **(*n* = 20)**		***p***
	**Baseline**	**Follow-up**	**Baseline**	**Follow-up**	
**Weight (Kg)**	104 ± 22	103 ± 22	105 ± 18	105 ± 18	0.172
**AC (cm)**	122 ± 13	119 ± 15	119 ± 12	118 ± 11	0.332
**BIM (Kg/m2)**	37 ± 6	36 ± 6	37 ± 5	37 ± 5	0.126
**SBP(mmHg)**	135 ± 21	133 ± 21	137 ± 21	121 ± 11^#^	0.028*
**DBP (mmHg)**	80 ± 10	83 ± 10	81 ± 10	77 ± 8^#^	0.007*
**HR (bpm)**	74 ± 10	76 ± 10	72 ± 7	68 ± 8^#^	0.034*
**HRV**					
VARR	2137 ± 1988	2060 ± 2065	2749 ± 2793	3621 ± 4092	0.279
RMSSD (ms)	34 ± 21	33 ± 24	41 ± 28	51 ± 41	0.181
LF abs (ms^2^)	666 ± 662	576 ± 666	772 ± 839	710 ± 846	0.889
HF abs (ms^2^)	595 ± 594	560 ± 677	896 ± 1362	1530 ± 2485	0.154
LF%	34 ± 6	36 ± 9	32 ± 7	26 ± 8^#^	0.037*
HF%	29 ± 14	28 ± 11	34 ± 14	43 ± 19^#^	0.046*
**SBPV**					
Variance	51 ± 34	32 ± 24	36 ± 28	33 ± 16	0.204

*AC* Abdominal circumference, *BMI* Body mass index, *SBP* Systolic blood pressure, *DBP* Diastolic blood pressure, *HR* Heart rate, *HRV* Heart rate variability, *VARR* Total variance of RR interval, *RMSSD* Square root of the mean of the square of successive differences between adjacent RR intervals, *LF abs* Low frequency spectral power, *HF abs* High frequency spectral power, *SBPV* Systolic Blood Pressure variability, *Variance* Total SBPV power

^#^*p* < 0.05

^*^p < 0.05

**Fig. 3:**
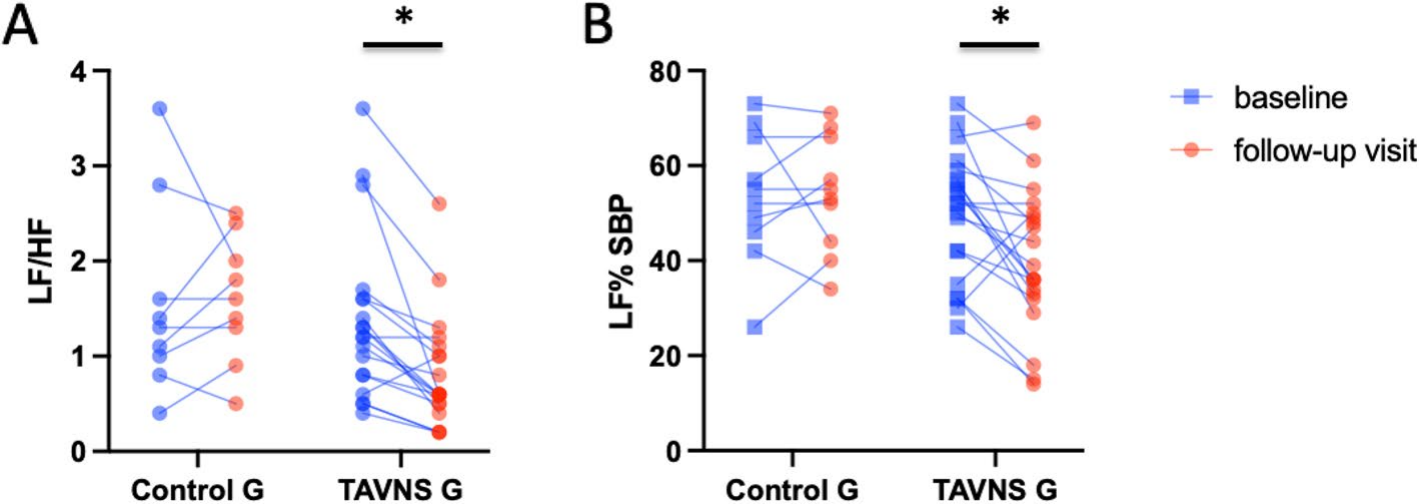
Autonomic balance in control and TAVNS groups at baseline and after 8 weeks of follow-up. **A** LF/HF ratio, *p = 0.026; **B** LF% SPB *p = 0.037

In addition, there were significant changes in components of the HRV in the frequency domain and in the systolic blood pressure variability, comparing baseline to follow-up in patients treated with TAVNS. After 8 sessions of TAVNS there was a significant increase in HF% of HRV, indicating an increase in vagal modulation. Moreover, there was a significant decrease in LF%, pointing to a reduction in sympathetic modulation (Table 3). Likewise, the LF/HF ratio, a marker of the sympathovagal balance, was significantly decreased in the treatment group (1.4 ± 0.9 vs 0.8 ± 0.6) but not in the control group (1.5 ± 1.0 vs 1.6 ± 0.6) (Fig. 3A). Additionally, the LF% component of the systolic blood pressure variability component, which suggests sympathetic modulation of the vasculature, decreased in patients that received TAVNS for 8 weeks (36 ± 13 vs 28 ± 13%) with no change in the control group. (27 ± 11 vs 30 ± 9%) (Fig. 3B).

To our knowledge, the present study is the first to investigate the effects of TAVNS once a week of for 8 weeks in MetS patients. The results indicate a beneficial TAVNS effect on cardiovascular autonomic function associated with a further decrease in blood pressure and heart rate. A previous study found that 15-min TAVNS administered every day during two weeks in healthy adults (> 55 years) was associated with increased vagally mediated HRV indexes and improved baroreflex sensitivity at rest. Participants with higher LF/HF at baseline showed a greater reduction in LF/HF at the end of the protocol (Bretherton et al. 2019).

In the current study, we observed a lower LF % component of SBPV, a variable that provides information about the contribution of sympathetic activity to vasomotor tone. Specifically, the reduction in LF% SBPV component detected in MetS patients points to reduced systemic sympathetic activity in patients receiving TAVNS. In agreement with this finding, muscle sympathetic nerve activity (MSNA) record is considered a direct measurement of sympathetic activity to the vessels and a previous study demonstrated a decrease MSNA recording in response to a 15 min-stimulation of the tragus of healthy subjects, which reinforces our finding (Clancy et al. 2014). Additionally, studies have indicated that acute TAVNS neuromodulation ameliorates left ventricular strain (Tran et al. 2019) and 6 months of VNS reduced atrial fibrillation events (Stavrakis et al. 2020). The antiarrhythmic activity may be a consequence of the TAVNS-mediated restoration of the tonic vagal inhibition over the hyperactivity of the intrinsic cardiac nervous system, ultimately resulting in an electrical and autonomic remodeling phenomenon (Carandina et al. 2021; Tran et al. 2019). In view of these observations, long-term TAVNS may be a therapeutic intervention to suppress systemic sympathetic activity and its potential adverse consequences on blood pressure.

### Chronic TANVS modulates circulating immune and endothelial cells

An example of FACS analysis is presented in Supplementary Figure 1. Five patients treated with TAVNS did not collect blood samples for this analysis at the end of follow-up, due to logistical problems or difficulties in sample collection. Thus, the study included 15 patients of TSNVS group and 10 controls (Supplementary Table 1). Technical issues during the FACS analysis caused missing data in few specific samples of cells.

**Fig. 4:**
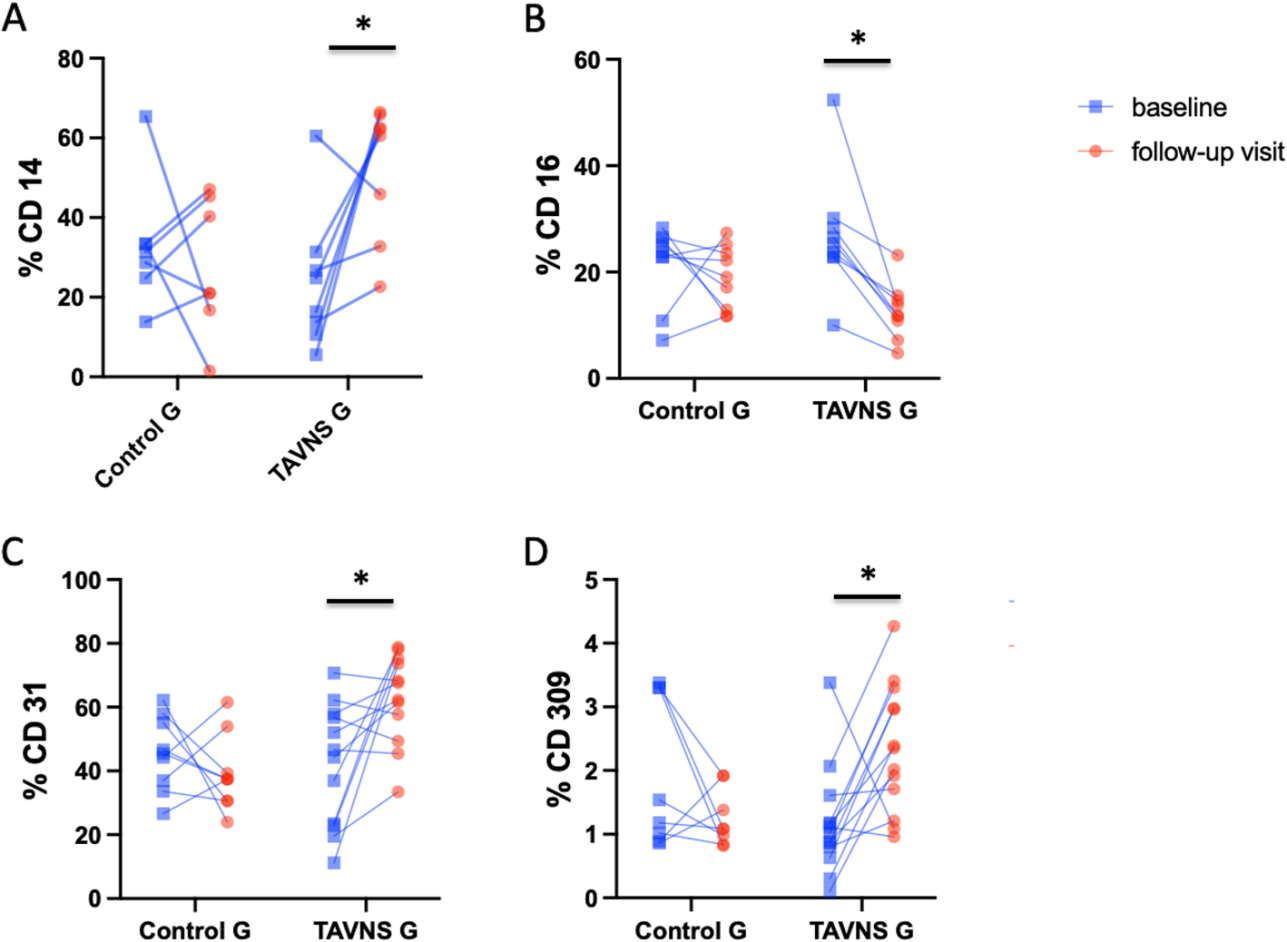
Circulating monocytes and endothelial cells percentages in control and TAVNS groups at baseline and after 8 weeks of follow-up. **A** CD14^+^ = classical monocytes (*p = 0.025) (**B**) CD16^+^ = non-classical monocytes (*p = 0.023) (**C**) CD31^+^ = circulating endothelial cells (*p = 0.015) (**D**) CD309.^+^ = endothelial progenitor cells (*p = 0.048)

Compared to baseline, patients treated with TAVNS exhibited a significant increase in percentage of classical monocytes (CD14^+^) and a significant decrease in of non-classical monocytes (CD16^+^) (Fig. 4A), which suggests a transition to a systemic anti-inflammatory monocyte profile. There was also a significant increase in the percentages of both circulating (CD31^+^) and progenitor endothelial cells (CD309^+^) (Fig. 4C, D) in the treated patients, suggesting that TAVNS modulates this linage to a reparative vascular profile. No significant alterations were observed in the control group (Fig. 4A, B, C, D).

The autonomic nervous system and the vagus nerve are critically implicated in the regulation of cytokine production, inflammatory processes, and immune cell migration (Lopes et al. 2016; Tracey 2002; Falvey et al. 2022; Pavlov and Tracey 2017; Pavlov and Tracey 2022; Carnevale et al. 2016; Carnevale et al. 2020; Fujimoto et al. 1994; Libby et al. 2016). In this study, there was a significant increase (three times) in the percentage of classical monocytes (CD14^+^) and a significant decrease (50%) in the percentage of non-classical monocytes (CD16^+^) after 8 sessions of TAVNS, while no changes were observed in the control group. These subpopulations have different immunological functions (Sprangers et al. 2016). The non-classical monocytes (CD16^+^—NCM) produce large amounts of interleukin (IL)-1β under baseline conditions or in response to stimulation with lipopolysaccharide (LPS), which led to the notion that NCMs exerts inflammatory functions most importantly in circulation (Yang et al. 2014). The production of IL-1 β is considered a marker of inflammatory activity in non-classical human monocytes. NCMs in obesity are in a pro-inflammatory state with an increase in intranuclear NF-kB binding, a decrease in IkB-beta (also designated as IKK2) and an increase in the transcription of pro-inflammatory genes regulated by NF-kappa B (Ghanim et al. 2004; Matos et al. 2016). On the other hand, classical monocytes (CD14^+^) participate in endothelial adhesion and cell migration, being considered to have an anti-inflammatory profile (Ghanim et al. 2004). The results of this study show that activation of a neural signaling with TAVNS can downregulate inflammatory cell phenotypes in blood, supporting the concept of TAVNS as a tool to quench proinflammatory mediators while triggering anti-inflammatory signaling.

Our understanding of monocyte subsets is currently limited to the distinction of three subsets in humans, but this is likely an oversimplification (Sprangers et al. 2016; Yang et al. 2014). While monocytes can differentiate into macrophages and dendritic cells under different conditions, it is not completely understood if there are specific monocyte subsets that give rise to specific subsets of macrophages or dendritic cells (Frodermann and Nahrendorf 2018). The current view is that under inflammatory conditions, monocytes are recruited to replace tissue resident macrophages which are lost via apoptosis. Inflammatory sites recruit monocytes mainly through the expression of specific chemokines. Classical monocytes have been thought to differentiate to inflammatory (M1) macrophages after recruitment, whereas non-classical monocytes differentiate to reparative (M2) macrophages. However, this is still a matter of debate (Frodermann and Nahrendorf 2018).

Within the adipose tissue, a critical contributor to the pathogenesis of MetS, macrophages have been shown to be important regulators of inflammation and metabolism (Ghanim et al. 2004; Matos et al. 2016). Activated classical macrophages (M1 macrophages) are involved in the initiation and maintenance of inflammation and are associated with the production of pro-inflammatory cytokines such as IL-1β, Il-6 and TNF. These cytokines produced by M1 macrophages in adipose tissue during obesity promote insulin resistance, which is a key feature of obesity and type 2 diabetes and other metabolic derangements (Odegaard and Chawla 2013). Non-classical macrophages (M2 macrophages) are involved in tissue repair and resolution of inflammation and are associated with the production of anti-inflammatory cytokines such as IL-10. These immune cells are typically found under normal conditions, and their activation is thought to be protective against the development of insulin resistance (Wentworth et al. 2010). The mechanisms underlying shifts in macrophage phenotype in MetS are not fully understood, but may involve a variety of factors, including changes in adipose tissue architecture, adipocyte dysfunction, and alterations in the gut microbiome (Olefsky and Glass 2010).

There is limited information about the role of the vagus nerve and the inflammatory reflex in macrophage polarization in the context of adipose tissue inflammation (Pavlov and Tracey 2017;  Wang et al. 2014). Further research will contribute to revealing potential therapeutic implications of VNS for modulating macrophage polarization in the context of adipose tissue inflammation and metabolic diseases.

Of note, subjects with MetS treated with 8 weeks of TANVS had an increase in the mean percentage of endothelial progenitor cells (EPCs) compared with baseline (100%), while no significant changes were detected in the control group. EPCs circulate in the blood and appear to reside preferentially at sites of vascular or tissue damage, contributing significantly to re-reendothelialization and physiological neoangiogenesis (Werner et al. 2005). There is a direct relationship between the EPC and peripheral endothelial function, indicating that the presence of EPCs may be a mechanism used to recover the dysfunctional endothelium (Schmidt-Lucke et al. 2005). The findings suggest that the improvement in the sympathovagal balance altered the hemopoietic release of cells, favoring vascular repair. We did not find a significant alteration in the percentage of endothelial microparticles (Supplementary Table 1).

There are some limitations of the present study. Regarding the protocol design, we did not use a simulated procedure in the control group, which would give more strength in the comparison between groups. Moreover, there is evidence that stimulation frequency and intensity can affect the extent of VNS. We applied TAVNS once a week. Although we detected significant improvement in frequency domain components of HRV and a decrease in hemodynamic parameters with this treatment, a daily stimulation could cause a stronger cardiovascular autonomic modulation. Nevertheless, it should be noted that TAVNS effects could last longer, even days, after the intervention, and perhaps could have a cumulative effect. Future studies should consider including a sham control group and increasing the frequency of stimulation of TAVNS. More importantly is the small number of patients included in the FACS analysis. Not all TAVNS-treated patients had blood samples for this analysis at the end of follow-up, due to logistical problems or difficulties in the sample collection. In addition, technical issues caused missing data on some specific cell samples. The small sample size increases the risk of type II error. However, the paired analysis showed a very homogeneous response in cell behavior in the treated group, which allowed finding significant differences between groups. Future studies including a larger number of patients are needed to confirm these results.

## Conclusion

For the first time, we have shown that TAVNS administered every week for eight weeks improves autonomic neural regulation in subjects with MetS. In addition, TAVNS decreases blood pressure, down regulates inflammatory cell phenotypes in blood, shifting towards an anti-inflammatory cellular milieu and up regulates endothelial cells to a reparative vascular profile. Therefore, our findings support the future exploration of TAVNS as a simple, non-invasive, and economical approach for improving the sympathovagal balance and potentially the quality of life for patients afflicted by a broad range of cardiovascular conditions.

## Supplementary Information

**Additional file 1: Supplementary Figure 1.** Example of FACS gate used for monocytes and endothelial cells. The 30,000 events were acquired to analysis. (A) Monocytes gate from forward scatter (FSC) and side scatter (SSC). A1=negative monocytes (CD14-CD16-); A2=classical monocytes (CD14+CD16-) and A3=non classical monocytes (CD14-CD16+) expression (histograms). (B) We used beads from FSC and SSC that are endothelial cells similar size (endothelial cells gate). This gate is the first step for the characterization endothelial circulating cells (CD31); endothelial progenitor cells (CD309, and endothelial microparticles (C31+CD144+). B1=CD31+CD144+ dot plot graph of double-staining gate Q2-2; B2=CD309+ dot plot graph of staining gate Q4-2.

**Additional file 2: Supplementary Table 1.** Description of antibodies used in the marking of endothelial cells and immune cells. **Supplementary Table 2.** Comparison of monocytes and endothelial cells of control and TAVNS groups at baseline and after 8-weeks of follow-up.

## Data Availability

All data generated are included in this article and its supplementary information files. The datasets analyzed during the current study are available from the corresponding author on reasonable request.

## Abbreviations

MetS: Metabolic syndrome
TAVNS: Transcutaneous auricular vagus nerve stimulation
HRV: Heart rate variability
BP: Blood pressure
HR: Heart rate
CVD: Cardiovascular diseases
VNS: Vagus nerve stimulation
PBMC: Peripheral blood mononuclear cell
TG: Treatment group
CG: Control group
LF: Low frequency
HF: High frequency
AC: Abdominal circumference
BMI: Body mass index
SBP: Systolic blood pressure
DBP: Diastolic blood pressure
HDL: High density lipoproteins
LDL: Low density lipoproteins
AST/GOT: Aspartate aminotransferase
ALT/GPT: Alanine aminotransferase
TSH: Thyroid-stimulating hormone
T4: Thyroxine
CRP: High sensitivity C reactive protein
HOMA-IR: Homeostatic model assessment index
VARR: Total variance of RR interval
RMSSD: Square root of the mean of the square of successive differences between adjacent RR intervals
SBPV: Systolic Blood Pressure variability
MSNA: Muscle sympathetic nerve activity
EPCs: Endothelial progenitor cells
