# Supplementary material for: Brief periods of transcutaneous auricular vagus nerve stimulation improve autonomic balance and alter circulating monocytes and endothelial cells in patients with metabolic syndrome: a pilot study
Source: Bioelectron Med. 2023 Mar 31;9:7. doi: 10.1186/s42234-023-00109-2 (PMC10064781; doi:10.1186/s42234-023-00109-2)
Supplement: Supplementary file 1 — Additional file 1: Supplementary Figure 1. Example of FACS gate used for monocytes and endothelial cells. The 30,000 events were acquired to analysis. (A) Monocytes gate from forward scatter (FSC) and side scatter (SSC). A1=negative monocytes (CD14-CD16-); A2=classical monocytes (CD14+CD16-) and A3=non classical monocytes (CD14-CD16+) expression (histograms). (B) We used beads from FSC and SSC that are endothelial cells similar size (endothelial cells gate). This gate is the first step for the characterization endothelial circulating cells (CD31); endothelial progenitor cells (CD309, and endothelial microparticles (C31+CD144+). B1=CD31+CD144+ dot plot graph of double-staining gate Q2-2; B2=CD309+ dot plot graph of staining gate Q4-2. [file 42234_2023_109_MOESM1_ESM.docx]

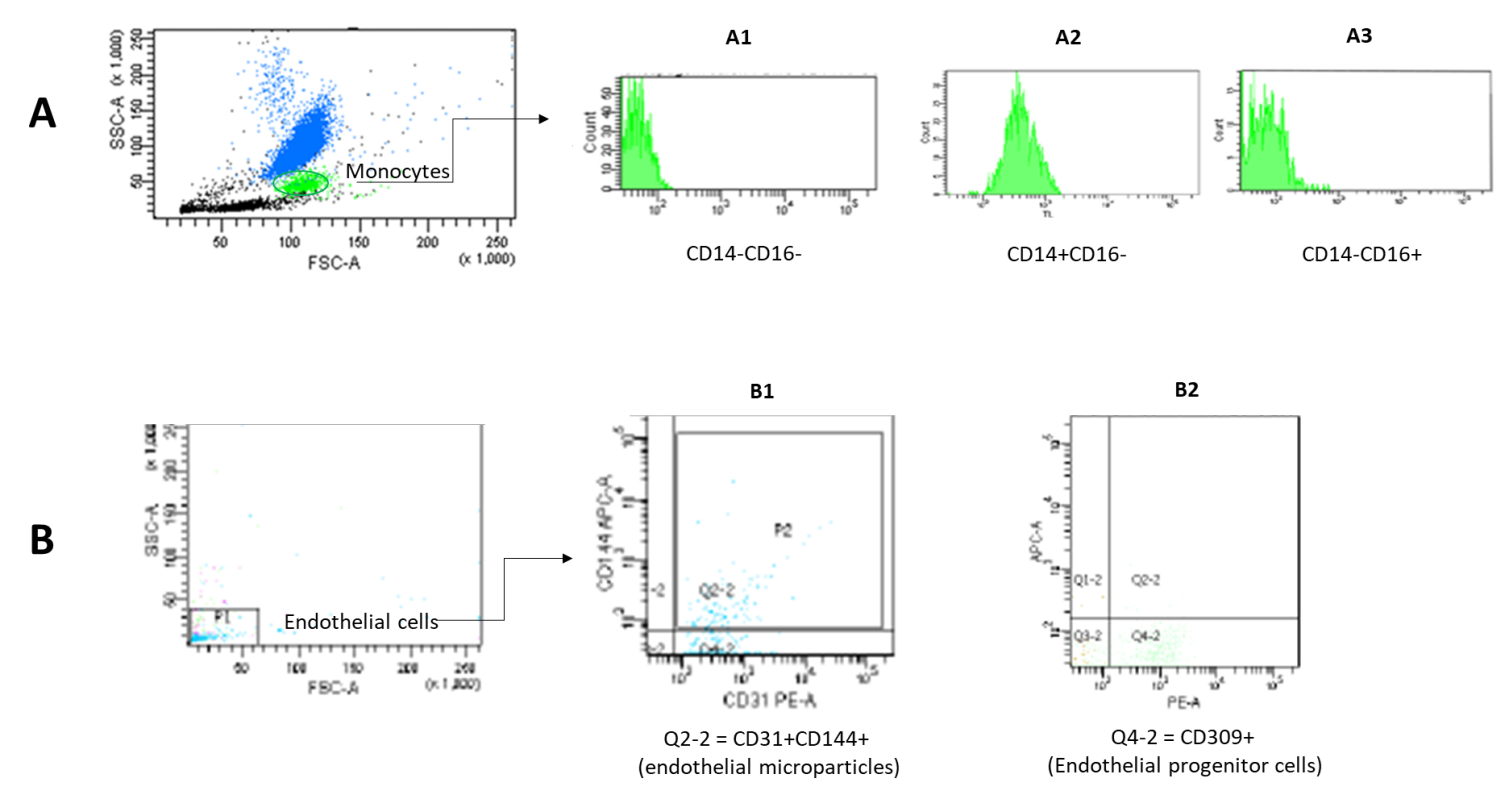


**Supplementary Figure 1**. **Example of FACS gate used for monocytes and endothelial cells.** The 30,000 events were acquired to analysis. (A) Monocytes gate from forward scatter (FSC) and side scatter (SSC). A1=negative monocytes (CD14^-^CD16^-^); A2=classical monocytes (CD14^+^CD16^-^) and A3=non classical monocytes (CD14^-^CD16^+^) expression (histograms). (B) We used beads from FSC and SSC that are endothelial cells similar size (endothelial cells gate). This gate is the first step for the characterization endothelial circulating cells (CD31); endothelial progenitor cells (CD309, and endothelial microparticles (C31^+^CD144^+^). B1=CD31^+^CD144^+^ dot plot graph of double-staining gate Q2-2; B2=CD309+ dot plot graph of staining gate Q4-2.
