# Supplementary material for: Brief periods of transcutaneous auricular vagus nerve stimulation improve autonomic balance and alter circulating monocytes and endothelial cells in patients with metabolic syndrome: a pilot study
Source: Bioelectron Med. 2023 Mar 31;9:7. doi: 10.1186/s42234-023-00109-2 (PMC10064781; doi:10.1186/s42234-023-00109-2)
Supplement: Supplementary file 2 — Additional file 2: Supplementary Table 1. Description of antibodies used in the marking of endothelial cells and immune cells. Supplementary Table 2. Comparison of monocytes and endothelial cells of control and TAVNS groups at baseline and after 8-weeks of follow-up. [file 42234_2023_109_MOESM2_ESM.docx]

**Supplementary Table 1.** Description of antibodies used in the marking of endothelial cells and immune cells

| **Marker** | **Concentration** | **Fluorochrome** | **Manufacturer/Catalog** |
| --- | --- | --- | --- |
| **CD31** | 2µL | PE | BD – 555446 |
| **CD144** | 2 µL | APC | BD – 561567 |
| **CD309** | 2 µL | PE | BD – 560494 |
| **CD14** | 2 µL | FITC | BD – 560996 |
| **CD16** | 2 µL | APC CY7 | BD - 557831 |

**Supplementary Table 2**. Comparison of monocytes and endothelial cells of control and TAVNS groups at baseline and after 8-weeks of follow-up.

| **Percentage** | **Control**  **(n=10)** | | **TAVNS**  **(n=15)** | |
| --- | --- | --- | --- | --- |
|  | **baseline** | **Follow-up** | **baseline** | **Follow-up** |
| **CD14^+^** | 33 ± 16 | 28 ± 17 | 24 ± 17 | 52 ± 17* |
| **CD16 ^+^** | 21 ± 7 | 19 ± 6 | 27 ± 11 | 13 ± 5* |
| **CD31^+^** | 45 ± 12 | 39 ± 12 | 42 ± 19 | 63 ± 14* |
| **CD309 ^+^** | 1.9 ± 1.2 | 1.3 ± 0.4 | 1.2 ± 0.9 | 2.4 ± 1.0* |
| **CD31^+^CD144^+^** | 4.9±4.3 | 3.0±2.9 | 3.5±1.6 | 3.2±1.0 |

CD14^+^=classical monocytes; CD16^+^=non-classical monocytes; CD31^+^=circulating endothelial cells; CD309^+^=endothelial progenitor cells; CD31^+^CD144^+^=endothelial microparticles; ******p* ≤ 0.05 compared to baseline
